# Liberation and Micellarization of Carotenoids from Different Smoothies after Thermal and Ultrasound Treatments †

**DOI:** 10.3390/foods8100492

**Published:** 2019-10-14

**Authors:** Magdalena Buniowska, Eva Arrigoni, Agata Znamirowska, Jesús Blesa, Ana Frígola, María J. Esteve

**Affiliations:** 1 Department of Dairy Technology, Faculty of Biology and Agriculture, University of Rzeszów, ul. Ćwiklinskiej 2D, 35-601 Rzeszów, Poland; mbuniowska@ur.edu.pl (M.B.); aznam@univ.rzeszow.pl (A.Z.); 2 Agroscope, Institute of Food Sciences, Schloss 1, CH-8820 Wädenswil, Switzerland; 3 Nutrition and Food Science, University of Valencia, Avda. Vicent Andrés Estellés, s/n, 46100 Burjassot, Spain; jesus.blesa@uv.es (J.B.); ana.frigola@uv.es (A.F.)

**Keywords:** ultrasound, intensive heat treatment, mild heat treatment, bioaccessibility, α-carotene, β-carotene, lutein, β-cryptoxantin

## Abstract

The consumption of a varied diet rich in fruit and vegetables helps prevent and treat certain chronic diseases. The development of smoothies based on derivatives from fruit and vegetables rich in bioactive compounds can help increase the consumption of these foods, and therefore, contribute to the prevention of various health problems. However, during the processing of the fruit and vegetable smoothies, these properties may change. The elaboration of smoothies is based on fruits and vegetables rich in carotenoids: Carrot juice-papaya-mango (smoothie A) and carrot juice-pumpkin-mango (smoothie B). The objective of this study is to evaluate the impact of the application of different thermal technologies (mild and intensive heat treatment) and non-conventional technologies (ultrasound) on carotenoids (α-carotene, β-carotene, lutein and β-cryptoxantin) and determine the physiochemical parameters of derivatives from fruit and vegetable smoothies. In addition, the bioaccessibility of carotenoids is also evaluated through a process of in vitro simulated digestion. With regard to the bioaccessibility of the fruit and vegetable smoothies analyzed, a positive effect of temperature on liberation and micellarization was observed.

## 1. Introduction

The yellow, orange, and red colorations of fruits and vegetables are usually caused by a large group of fat-soluble pigments called carotenoids. Low consumption of carotenoids is negatively related to a higher risk of cardiovascular diseases, cancer, cataracts, and age-related macular degeneration. It has been hypothesized that this inverse relationship is caused by the antioxidant capabilities of carotenoids [1,2]. It is important to obtain more information about the bioaccessibility of carotenoids from foods in order to achieve a better understanding of their potential benefits.

One way of increasing fruit and vegetable intake is to drink smoothies—blended beverages made of fruit, fruit pulp, fruit juices, vegetables, yogurt and milk. In order to extend their shelf-life, smoothies are often thermally processed. Many studies have demonstrated that this process can affect the color of foods [3]. Bioactive compounds like carotenoids must be released from the matrix and reach their site of action to exert their biological effects, so bioaccessibility and bioavailability are critical features in assessing the role of these compounds in human health. Bioaccessibility represents the maximum number of carotenoids released from the food that is available for absorption in the enterocytes, while the fraction of the dose entering the systematic circulation to participate in physiological function is called bioavailability [4]. Nowadays, there is no clear consensus on the best approach to estimate carotenoid bioaccessibility. The application of in vitro digestion and assimilation procedures is necessary to gain information about factors that modulate different steps of carotenoid liberation and micellarization [5,6].

Liberation is defined as the percentage of carotenoids transferred during digestion from the test smoothies to the aqueous micellar fraction and has been a source of recent research interest. In contrast to liberation, micellarization describes only the percentage of carotenoids transferred during in vitro digestion to micelles after ultracentrifugation. Micellarization is, therefore, a part of accessibility, which indicates how efficiently a released carotenoid is incorporated into mixed micelles [7].

There are many studies available that have investigated the in vitro absorption of carotenoids using in vitro digestion methods. The liberation and micellarization of carotenoids can be affected by a variety of factors, including the amount ingested, food source, matrix, amount and type of processing, amount and type of fat co-ingested, carotenoid interactions, and other dietary components, such as fiber [8,9,10].

The conventional thermal processing of fruit and vegetable smoothies remains the most widely-adopted technology for shelf-life extension and preservation of these products. However, consumer demand for fresh and nutritious juices has led to an interest in non-thermal technologies to apply in the processing of fresh vegetable smoothies to avoid the deleterious effects that heat has on the flavor, color and nutrients [11]. Non-thermal methods, such as ultrasound treatment have been proposed as alternatives to thermal mild heat treatment so that the changes to flavor and nutritional value can be minimized during processing.

The aim of this study is to compare the effect of thermal treatment (mild and intensive heat treatment) with non-conventional technology (ultrasound), from the point of view of the food safety, quality and preservation of nutritional compounds (carotenoids), and to evaluate the in vitro bioaccessibility of carotenoids in smoothies.

## 2. Reagents and Methods

### 2.1. Reagents

Potassium Chloride (KCl), Sodium Chloride (NaCl), Magnesium Chloride Hexahydrate (MgCl_2_(H_2_0)_6_), and Sodium Hydroxide (NaOH) were purchased from FlukaChemie AG (Buchs, CH, Switzerland). Potassium Dihydrogen Phosphate (KH_2_PO_4_), Sodium Hydrogen Carbonate (NaHCO_3_), 2,6-Di-*tert*-butyl-4-methylphenol, Butylated Hydroxy toluene (BHT) were purchased from Sigma-Aldrich (Buchs, CH, Switzerland). Calcium Chloride Hexahydrate (CaCl_2_(H_2_O)_6_) was obtained from Merck Millipore. Acetone (analytical grade), Sodium Hydroxide (NaOH), Methanol and Acetonitrile were purchased from Carlo ErbaReagentiSpA (Rodano, Italy). HPLC-grade Dichloromethane and Hydrogen Chloride (HCl) were purchased from Burdich and Jackson (Seelze, Germany).

Digestive enzymes (Alpha-Amylase from human saliva, pepsin from porcine and from gastric mucosa, and pancreatin from the porcine pancreas) and porcine bile from bovine and ovine were purchased from Sigma-Aldrich (Buchs, CH, Switzerland).

All high purity standards (96–98%), including β-carotene, α-carotene, β-cryptoxantin, and lutein were purchased from Carote Nature GmbH (Ostermundigen, CH, Switzerland).

### 2.2. Sample Preparation

The elaboration of smoothies is based on fruits and vegetables rich in carotenoids: Carrot juice-papaya-mango (smoothie A) and carrot juice-pumpkin-mango (smoothie B).

Pumpkin, papaya, mango, and skimmed milk (0.1% fat) were purchased from a supermarket in Poland (Tesco, Rzeszow, Poland). Carrot juice was purchased from Witmar (Rzeszow, Poland). Three different samples were prepared. The fruits were washed, dried and chopped in a blender for 5 min. Smoothie A was obtained by mixing carrot juice (20%) with citrus pectin (0.3%, *w*/*v*), papaya (30%) (*v*/*v*) and mango (50%) (*v*/*v*). Smoothie B was prepared based on carrot juice (20%), pumpkin (30%) and mango (50%). After blending, all smoothie samples were transferred to 250 mL bottles. A fresh control sample was chilled (2–4 °C) immediately, while other samples were subjected to subsequent processing. 

### 2.3. Microbial Analysis

Samples were taken before and after filling, from the raw and different types of treated smoothies. A total of 10 g of each sample was mixed with 90 mL peptone solution. Serial dilutions from 10^−1^ to 10^−9^ were carried out, and the microbial status was evaluated by means of plate counts. To determine the total variable bacterial, agar Plate Count Agar (Oxoid Ltd., Basingstoke, UK) was used and incubated at 30 °C for 72 h. Next, the grown bacterial colonies were counted. The total number of bacteria per gram of smoothies was obtained by multiplying the number of colony-forming units (CFU) on the plate with the respective dilution factor, and this was then converted into logarithmic form. *Enterobacteriaceae faecalis* were enumerated using a Slanetz and Bartley agar (Oxoid, Basingstoke, UK) surface and incubated at 37 °C for 48 h. Next, the grown bacterial colonies were counted. For the inoculation of yeast and mold, chlorofenicol agar (Oxoid, Basingstoke, UK) was used and incubated at 25 °C for five days. All samples were analyzed in triplicate. All measurements were expressed in cfu/mL (colony-forming units per milliliter of the sample).

### 2.4. Smoothie Color Measurement

Color was measured in duplicate using a spectrophotometer Minolta CM-2002, Konica Minolta Ewing, (NJ). The results were expressed in accordance with the Commission International d`Eclairage LAB (CIELAB) system. Two consecutive measurements of each sample were taken. The L* (lightness (0 = black, 100 = white); a* (−a* = greenness, +a* = redness); and b* (−b* = blueness, +b* = yellowness) were measured [12].

### 2.5. In Vitro Digestion Model

An in vitro digestion procedure based on the harmonized Infogest protocol [13] and slightly modified from Kopf-Bolanz et al. [14] was used. In vitro gastrointestinal digestion was carried out in two sequential phases: Gastric and intestinal digestion. Approximately 5 g of the sample was weighed in triplicate and added to 50 mL brown bottles. Then, 10 mL of gastric fluid (porcine pepsin 0.39 mg/mL order nr. P7012, CaCl_2_(H_2_O)_2_ 0.15 μL/mL) was added to the samples. Gastric digestion was simulated by lowering the pH to 2 using 1 mol L^−1^ HCl. The sample was covered with nitrogen (for approximately 20 s) and incubated in a shaking (90 rpm) water bath for 2 h at 37 °C. After 2 h, intestinal digestion was initiated by adding 20 mL of duodenal juice, which contained bovine and ovine bile (0.0167 g/mL order nr. 8631, CaCl_2_(H_2_O)_2_ 0.15 μL/mL, pancreatin 0.011 g/mL order nr. P754). The pH was then adjusted, if necessary, to 7.0 ± 0.2. Samples were covered with nitrogen and incubated for 2 h as above. After 2 h, the digested samples were transferred to 50 mL tubes and centrifuged for 10 min at full speed (4495 rpm) at ≤5 °C (Heraeus Multifuge 3RS+, Thermo Scientific) to separate the solids from the aqueous phase. For the quantification of carotenoid liberation, 5 mL of the aqueous supernatant was freeze-dried immediately under exclusion of light and oxygen, whereas, for in vitro accessibility (micellarization) analysis, aliquots were filtered through 0.22 lm syringe filters before freeze-drying [15]. Exactly 1 mL of the filtered in vitro accessibility fraction was freeze-dried.

All samples were frozen in liquid nitrogen under constant rotation. Then, the samples were freeze-dried (Christ Alpha 2–4 LSC, Adolf Kühras AG, Basel, CH, Switzerland) overnight or until dryness. The in vitro digestion was performed in triplicate.

### 2.6. UHPLC Analyses

Carotenoid identification and quantification were analyzed using Ultra-High-Performance Liquid Chromatograph Acquity UPLC^®^ H-Class Bio (Agilent Technologies, Santa Clara, CA, USA) with a Van Guard C18 column 2.1 × 50 mm (with a particle size of 1.8 μm) ((Waters), AG, Baden-Dättwil, CH, Switzerland), pre-column Acquity UPLC C18 1.8 µm, Vanguard Pre-Column 3/pk, 2.1 × 5 mm column (Waters)) and a photodiode array detector (PDA) with a range of 190–500 and a sensitivity range of 0.0001–4.0000 AUFS. The gradient mobile phase solvent A was a mix of 25% 0.05 mol/L ammonium acetate and 75% acetonitrile (ACN): dichloromethane: methanol (MeOH) (75:10:15). Solvent B was a mix of acetonitrile (ACN)/dichloromethane/methanol (MeOH) (60:10:30). The following separation program was applied: 0–4.5 min: 100% A, 4.5–7.5 min: 100% A → 100%, B 7.5–12.5 min: 100% B, 12.5–16 min: 100% A, 16–19 min: 100% A. The flow rate was 0.45 mL/min, the injection volume was 5.0 μL, and the column temperature was maintained at 35 °C using a column oven. Autosample temperature: 4 °C. Detectio: 425, 450, and 470 nm. Peak identification was achieved by the individual injection of each pigment standard and by comparing spectra [15].

### 2.7. Extraction and Quantification of Carotenoids

Methanol and acetone (Acros Organics Chemie Brunschwig, Basel, Switzerland) with a purity of >99% were mixed 1:1 (*v*/*v*), and the extraction solvent was completed by adding 0.01% of 2,6-di-tert-butyl-methylphenol (Sigma-Aldrich Chemie GmbH, Buchs, Switzerland) per liter. For extraction, aliquots (5 g) of the sample were mixed with 50 mL of the extraction solvent, flushed with nitrogen for 30 s and concomitantly homogenized with a Polytron PT 3100, MERCK (Zug, Switzerland). Then, a vortex was briefly used, and an ultrasonic bath was used for 30 min. Samples were left to sediment for ≥5 min and then filtered through 0.22 μm nylon filters directly into UPLC vials. 

For UPLC analyses after in vitro digestion, the extraction solvent (MeOH: Acetone 1:1 containing 0.01%BHT) was added to the freeze-dried samples in glass bowls: In general, in a 1:1 ratio, i.e., 5 mL to pear-shaped flasks (for liberation) and 1 mL to hydrolysis tubes (for micellarization). Then, a vortex was briefly used, and an ultrasonic bath was used for 30 min. Samples were left to sediment for ≥5 min and then filtered through 0.22 μm nylon filters directly into UHPLC vials.

To quantify the carotenoid concentrations, regression lines were calculated for each carotenoid standard. In all cases, the regression lines were based on injecting different concentration levels from 0.025 up to 3.75 mg/L.

Liberation was considered as the concentration of carotenoid content released from the food matrix by in vitro gastrointestinal digestion and that which is available for absorption. Liberation was calculated using Equation (1):

Liberation (%) = (supernatant/starting material) × 100,(1)

where the supernatant and starting materials corresponded to the carotenoid concentration mg/100 g in the dialyzed fraction and non-digested smoothie, respectively.

Micellarization is defined as the percentage of carotenoid transferred from the digestate to micelles after centrifugation and microfiltration. In vitro micellarization was calculated as the percentage of the respective carotenoid transferred from the test food to the micellar phase obtained by microfiltration of the supernatant.

### 2.8. Treatments

Liquid foods have traditionally been preserved by thermal treatments, which is the used method used to extend the shelf-life of liquid foods by preventing microorganism spoilage and contamination with pathogens. However, this treatment leads to the loss of nutritional compounds and undesirable changes in the sensory properties of food [16,17]. For our study, total viable counts and yields and mold counts were analyzed for all samples before and after the treatments were applied. The initial microbial concentration in smoothies was approximately 10^4^ cfu/mL.

Different conditions of mild and intensive heat treatment, and ultrasound treatment are studied, choosing conditions in which no bacterial growth or colonies are observed. For the different thermal treatments applied, temperatures of 90 °C for mild heat treatment and 120 °C for intensive heat treatment were tested at a holding time of 20 s using autoclave units (Autester 18B). For the ultrasound treatment, an ultrasonic Bracket bath with a continuous wave of 60 °C for 20 min was used. After the treatments, all smoothie samples were immediately cooled down in refrigeration temperatures of 2–4 °C.

### 2.9. Statistical Analysis

Results were expressed as the mean ± standard deviation of the determination. An analysis of the variance (ANOVA) of the results was carried out in order to determine significant differences (*p* < 0.05) between the different treatments and the concentration of carotenoid in non-digested smoothies and that obtained in the different phases of the in vitro gastrointestinal digestion. For all samples, carotenoid analyses were performed at least in triplicate (*n* = 3–5). All data were examined with SPSS 24.0 for Windows (SPSS Inc.; Chicago, IL, USA).

## 3. Results and Discussion

### 3.1. Undigested Samples, Immediately after Thermal and Ultrasound Treatments

For each sample, three different carotenoids were quantified. In smoothie A, based on carrot juice-papaya-mango, β–cryptoxanthin, α–carotene and β–carotene were detected. In smoothie B, based on carrot juice-pumpkin-mango, lutein, α–carotene and β–carotene were detected.

Much research has studied the effects of different thermal and non-thermal methods on the levels of carotenoids in food; some report substantial losses, others no change, while others found increased carotenoid content [18,19].

Table 1 shows the individual carotenoid content in smoothies treated by thermal and ultrasound treatments in comparison with untreated samples. As we can observe, there were different carotenoid contents depending on the different treatments applied. A range of three identified carotenoids included β-cryptoxanthin (0.10–0.21 mg/100 g), α-carotene (1.66–1.98 mg/100 g) and β-carotene (2.24–2.74 mg/100 g) for smoothie A, and lutein (0.15–0.44 mg/100 g), α-carotene (1.53–1.86 mg/100 g) and β-carotene (2.09–3.02 mg/100 g) for smoothie B. In both smoothies, β-carotene was the most abundant carotenoid.

With regard to all treatments, a significant difference after intensive heat treatment in the content of β–cryptoxanthin and lutein was observed. According to a study performed by Lee and Coates [18], losses after pasteurization (90 °C and 30 s) in the content of the most labile xanthophylls were detected. However, in our study, no significant changes were observed after the mild heat treatment of both smoothies. On the other hand, no significant changes were observed after intensive and mild heat treatment in β-carotene. The results are in agreement with those found by Patras et al. [19] for thermally processed (70 °C, 120 s) strawberry and blackberry purées and by Barba et al. [20] who applied thermal processing (90 °C for 15 and 21 s, and 98 °C for 15 and 21 s) in an orange juice-milk beverage. The concentration of α-carotene decreases during the intensive heat treatment process in both smoothies, although the changes are only significant in smoothie B.

Moreover, there were no significant changes observed in carotenoids after ultrasound treatment except in β-carotene content in smoothie B, where a slight increase after ultrasound treatment was detected. This phenomenon can be explained by the ability of ultrasound to enhance the disruption of cell walls, which might have facilitated the release of bound carotenoid content. In this line, Abid et al. [21] found an improvement in carotenoid content after ultrasound treatment in apple juices. Moreover, Rawson et al. [22] found a slight increase in lycopene content after ultrasound treatment in watermelon juice.

### 3.2. Colour Analysis

Carotenoids are known as pigments responsible for many colors of leaves, fruits, and flowers in plants. The color depends on their growth and maturity and the concentration of carotenoid isomers. The presence of various pigments, such as α-carotene, β-carotene, β-cryptoxanthin, and lycopene, and unresolved mixtures of pigments is responsible for the color of fruits and vegetables. Carotenoids are heat stable in systems with minimum oxygen content. However, high carotenoid foods may change color under the influence of thermal processing, because heat induces cis-trans isomerization reactions. Colorimetric measurements of smoothies prior to both thermal and ultrasound treatment for smoothie A: 63.02, 34.13, and 47.02 for L* (lightness), a* (redness), and b* (blueness) parameters, respectively. For smoothie B, a higher value of b* parameters, 54.51, was observed (Table 2). With regard to CIELAB parameters, the statistical analysis of the results obtained after intensive heat treatment showed a significant difference (*p* < 0.05) for the b* values compared to untreated samples. This finding is supported by the investigation by Walkling-Ribeiro et al. [23] who found a significant decrease in b* parameters when mild pasteurization (72 °C, 15 s) was applied in fruits smoothies. Compared to the untreated smoothies, non-significant changes in a* values were found in both smoothies after each treatment was applied. Moreover, no significant change was observed after ultrasound treatment in all parameters. Additionally, a slight increase in L* in smoothie B was observed, but was non-significant. This is in agreement with prior investigations when Sengun et al. [24] observed an increase in lightness (L*), but a decrease in blue yellow (b*) in ultrasound-treated grape juice compared with non-treated juice. Furthermore, Zenker et al. [25] observed an increase in the lightness of ultrasound-treated orange juice. Moreover, Pala et al. [26] did not find significant changes in the redness (a*) of the juice after ultrasound treatment at an amplitude of 75% and 100% for up to 24 and 12 min, respectively. These results suggest that ultrasound treatment has potential uses as an alternative non-thermal technique for traditional thermal mild heat treatment processes for maintaining the quality of smoothies from fruit and vegetables.

### 3.3. Liberation and Micellarization of Carotenoid Content from Different Smoothies Subjected to Thermal Processing and Ultrasound Treatment

In order to determine the nutritional efficacy of the recovered compounds, quantification directly from foodstuff is not enough for the prediction of potential in vivo effects. Therefore, the determination of valuable compound bioaccessibility is necessary. In the present study, we estimate the liberation and micellarization of carotenoids from different smoothies to obtain an effective tool for the initial screening of carotenoid bioaccessibility.

**Figure 1:**
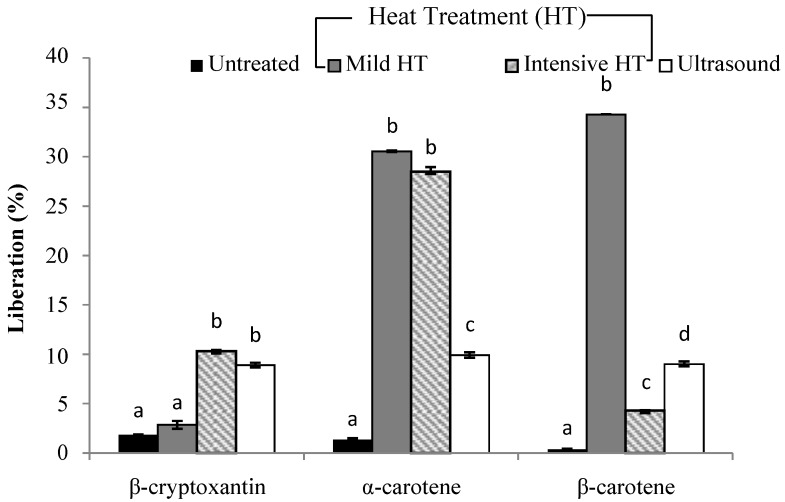
The efficiency of liberation (%) in carotenoids after the in vitro digestion of a smoothie based on carrot juice, papaya and mango (smoothie A). (**a**–**d**) Different letters in the same column group indicate a significant statistical difference as a result of the applied treatment (*p* > 0.05).

**Figure 2:**
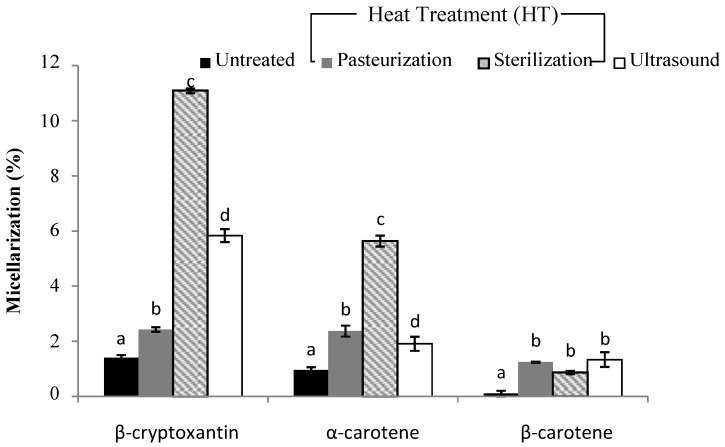
The efficiency of micellarization (%) in carotenoids after in vitro digestion of smoothie based on carrot juice, papaya and mango (smoothie A). (**a**–**d**) Different letters in the same column group indicates a significant statistical difference in the function of the applied treatment (*p* > 0.05).

**Figure 3:**
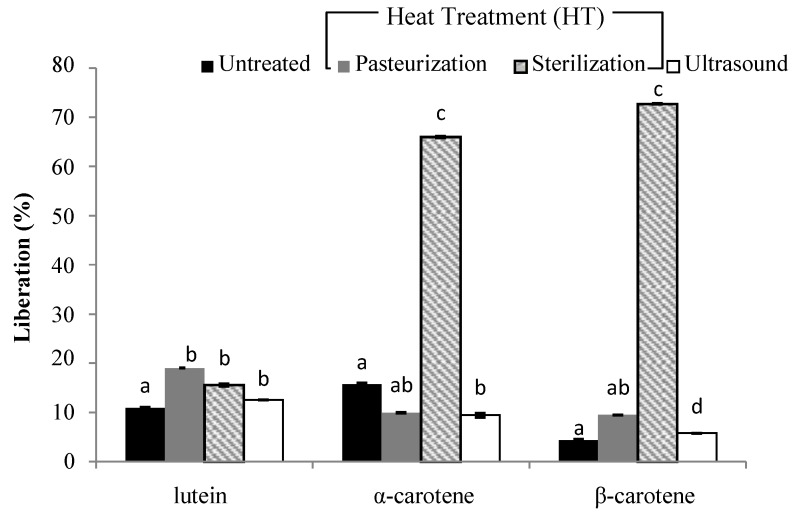
The efficiency of liberation (%) in carotenoids after in vitro digestion of a smoothie based on carrot juice, pumpkin and mango (smoothie B). (**a**–**d**) Different letters in the same column group indicate a significant statistical difference as a result of the applied treatment (*p* > 0.05).

**Figure 4:**
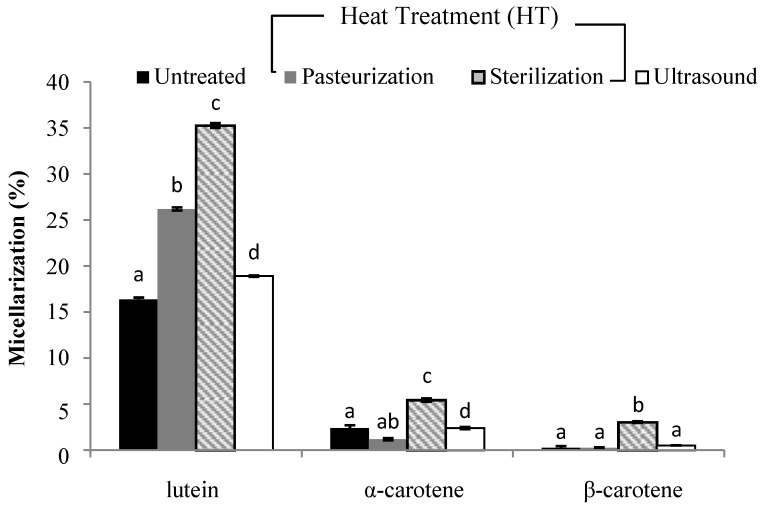
The efficiency of micellarization (%) in carotenoids after in vitro digestion of a smoothie based on carrot juice, pumpkin and mango (smoothie B). (**a**–**d**) Different letters in the same column group indicate a significant statistical difference as a result of the applied treatment (*p* > 0.05).

The analysis regarding carotenoid content during the simulated gastrointestinal in vitro digestion is shown in Figure 1, Figure 2, Figure 3 and Figure 4.

As shown below in Figure 1, a significant difference in carotenoid liberation after applying different treatments was observed, but the percentages were widely distributed. According to the experimental results achieved, all treatments led to an increase in carotenoid liberation in smoothie A in comparison with the untreated sample. This increase in carotenoid liberation after technical processes can be explained by the disruption of the natural matrix during food processing. β-cryptoxanthin liberation was in the range of 3%, 10.3%, and 9% for mild and intensive heat treatment, and ultrasound treatment, respectively. The highest liberation (%) was obtained for β-carotene after mild heat treatment (34.2%). This result is in line with the study performed by Lemmens et al. [27] who found an improvement in β-carotene accessibility after thermal treatments. In this way, Knockaert et al. [28] revealed that mild heat treatment causes an increase in β-carotene liberation in carrot juice. Moreover, α-carotene liberation was enhanced significantly after both thermal treatments in comparison with the untreated sample. Furthermore, Aschoff et al. [7] observed a higher carotenoid liberation in pasteurized orange juice, 53.9%, in comparison with freshly squeezed juice, 44.9%. Another author, Stinco et al. [29], observed a difference in carotenoid liberation between fresh orange juice, where he detected 30%, and industrially squeezed and pasteurized juice, which had a liberation value of up to 52%. With regard to ultrasound treatment, the liberation of all carotenoids was in the range of 9 ± 0.2%. This may indicate that ultrasound waves during cavitation destroy plant cell walls and cause the extraction of more bioactive compounds that enhance liberation. However, enhancement was not so high, which can be explained by the treatment condition (temperature 60 °C, over 20 min). In one study by Bengtsson et al. [30], an improvement of β-carotene after grinding treatments were applied was observed.

Regarding micellarization (%), a significant difference after applying different treatments was observed. The results are shown in Figure 2. Generally, all cases show a higher liberation value compared to bioaccessibility, and this is in accordance with a previous study which reported significantly lowered carotenoid levels after the microfiltration of the aqueous supernatant [31,32]. In our study, micellarization (%) was in the range of 0.4–11.09%. As we can observe, the value of β-carotene after the micellarization stage was limited in the unprocessed sample (<0.5%). A smoothie subjected to the intensive heat treatment showed significantly higher β-cryptoxanthin and α-carotene micellarization compared to the unprocessed raw smoothie. Although carotenoid retention may be negatively affected by thermal treatments, this does not necessarily affect their transfer to micelles. According to Fernández-García et al. [5], the degree of food processing is significant for the micellarization efficiency of carotenoids, as a high processing degree can maximize the amount of compound that is made soluble from the matrix. On the other hand, in the case of β-carotene, there was no significant difference observed in micellarization between different treatment applied. Our results are similar to those reported by Gupta et al. [33], who detected that micellarized β-carotene did not differ significantly between the treatments applied.

In terms of smoothie B (Figure 3), liberation (%) in all carotenoids after the different treatments were applied was in the range of 5.8% to 73%. Surprisingly, no significant difference was found between the quantities of lutein in the digesta compared with different treatments. Moreover, all three methods applied enhanced lutein micellarization (Figure 4). Regarding liberation, a significant increase was observed when intensive heat treatment was used in the range of 66% and 73% for α-carotene and β-carotene, respectively. This would imply that at the chromoplast level, different matrices with different compositions were modified differently upon processing depending on their structural characteristics.

Micellarization in smoothie B (Figure 4) was significantly lower for α-carotene and β-carotene in comparison with smoothie A and was in range of 1.1–5.4% and 0.2–3.1%, respectively. Mild heat treatment generally reduced (*p* < 0.01) β-carotene micellarization. The efficiency of micellarization may be dependent on the type of food matrix and/or the composition of the final digesta in which they are contained [34]. Only 0.5% of β-carotene in the untreated sample was observed in the micelles. Moreover, after mild heat treatment and ultrasound, a low percentage of α-carotene (1.1% and 2.4%) micellarization was observed. Furthermore, a lower percentage for β-carotene (0.2% and 0.5%) micellarization was detected. Lutein micellarization was in the range of 26.1%, 35.2% and 18.9% for mild and intensive heat treatment, sand ultrasound treatment, respectively. In comparison with other carotenoids, lutein showed a greater extent in the digest from smoothies after the treatments were applied. Generally, α-carotene and β-carotene were statistically less efficiently transferred to the micelles than lutein. This fact can be explained by the lower lipophilicity of lutein [35]. In relation to our results, Ryan et al. [36] also observed higher micellarization (%) in lutein after different thermal treatments were applied. In accordance with the study by Rich et al. [37], a big difference between lutein and other carotenoid micellarization is that lutein in vegetables is more soluble than β-carotene in the micelle fraction.

## 4. Conclusions

The consumption of smoothies, with a good combination of fruits and vegetables, will guarantee vitamins, minerals and bioactive compounds, such as carotenoids, as well as fiber, in the diet. An improved microbiological shelf-life was achieved in a fruit smoothie using thermal treatments (mild and intensive). The assessment of color was found to be slightly better for smoothies after ultrasound rather than thermal treatment. Although thermal treatment reduced the content of carotenoids in foods, a positive effect of liberation, micellarization, and therefore, bioaccessibility was found.

## Figures and Tables

**Table 1:** The carotenoid content of different smoothies before and after different treatments were applied.

	**Smoothie A: Carrot Juice-Papaya-Mango**			
	**Untreated**	**Intensive Heat Treatment**	**Mild Heat Treatment**	**Ultrasound**
β-Cryptoxanthin	0.21 ± 0.01 ^a^	0.10 ± 0.01 ^b^	0.15 ± 0.00 ^ab^	0.18 ± 0.01 ^a^
α-Carotene	1.98 ± 0.16 ^a^	1.66 ± 0.19 ^a^	1.88 ± 0.03 ^a^	1.92 ± 0.13 ^a^
β-Carotene	2.74 ± 0.24 ^a^	2.24 ± 0.25 ^a^	2.53 ± 0.05 ^a^	2.42 ± 0.10 ^a^
	**Smoothie B: Carrot Juice-Pumpkin-Mango**			
	**Untreated**	**Intensive Heat Treatment**	**Mild Heat Treatment**	**Ultrasound**
Lutein	0.44 ± 0.01 ^a^	0.15 ± 0.01 ^b^	0.27 ± 0.01 ^ab^	0.38 ± 0.01 ^ab^
α-Carotene	1.83 ± 0.09 ^a^	1.53 ± 0.18 ^b^	1.80 ± 0.04 ^a^	1.86 ± 0.08 ^a^
β-Carotene	2.82 ± 0.16 ^a^	2.09 ± 0.21 ^a^	2.39 ± 0.05 ^a^	3.02 ± 0.09 ^b^

^a,b^ Different lowercase letters in the same file indicate a significant statistical difference as a result of the applied treatment (*p* > 0.05).

**Table 2:** Color changes of different smoothies before and after different treatments applied.

	**Smoothie A: Carrot Juice-Papaya-Mango**			
	**Untreated**	**Intensive Heat Treatment**	**Mild Heat Treatment**	**Ultrasound**
L*	63.02 ± 0.03 ^a^	60.08 ± 0.13 ^a^	59.33 ± 0.07 ^ab^	59.66 ± 0.05 ^a^
a*	34.13 ± 0.11 ^a^	32.04 ± 0.05 ^ab^	34.15 ± 0.07 ^a^	33.60 ± 0.41 ^a^
b*	47.02 ± 0.02 ^a^	34.11 ± 0.15 ^b^	47.14 ± 0.05 ^a^	44.50 ± 0.28 ^ab^
	**Smoothie B: Carrot Juice-Pumpkin-Mango**			
	**Untreated**	**Intensive Heat Treatment**	**Mild Heat Treatment**	**Ultrasound**
L*	63.02 ± 0.03 ^a^	59.08 ± 0.13 ^a^	59.33 ± 0.07 ^a^	64.66 ± 0.05 ^ab^
a*	32.14 ± 0.14 ^a^	31.81 ± 0.18 ^a^	32.17 ± 0.18 ^a^	31.22 ± 0.29 ^a^
b*	54.51 ± 0.44 ^a^	50.10 ± 0.09 ^b^	51.78 ± 0.01 ^ab^	55.20 ± 0.12 ^a^

L* lightness. a* redness. b* yellowness. ^a,b^ Different lowercase letters in the same file indicate a significant statistical difference, as a result of the applied treatment (*p* > 0.05).
